# Swinging a sword: how microtubules search for their targets

**DOI:** 10.1007/s11693-014-9134-x

**Published:** 2014-02-16

**Authors:** Nenad Pavin, Iva M. Tolić-Nørrelykke

**Affiliations:** 1 Department of Physics, Faculty of Science, University of Zagreb, Bijenička 32, 10000 Zagreb, Croatia; 2 Max Planck Institute of Molecular Cell Biology and Genetics, Pfotenhauerstrasse 108, 01307 Dresden, Germany; 3 Division of Molecular Biology, Ruđer Bošković Institute, Bijenička 54, 10000 Zagreb, Croatia

**Keywords:** Microtubules, Kinetochores, Mitosis, Search mechanism, Pivoting, Angular movement

## Abstract

The cell interior is in constant movement, which is to a large extent determined by microtubules, thin and long filaments that permeate the cytoplasm. To move large objects, microtubules need to connect them to the site of their destination. For example, during cell division, microtubules connect chromosomes with the spindle poles via kinetochores, protein complexes on the chromosomes. A general question is how microtubules, while being bound to one structure, find the target that needs to be connected to this structure. Here we review the mechanisms of how microtubules search for kinetochores, with emphasis on the recently discovered microtubule feature to explore space by pivoting around the spindle pole. In addition to accelerating the search for kinetochores, pivoting helps the microtubules to search for cortical anchors, as well as to self-organize into parallel arrays and asters to target specific regions of the cell. Thus, microtubule pivoting constitutes a mechanism by which they locate targets in different cellular contexts.

## Introduction

A living cell is never at rest; organelles and molecular assemblies constantly move in its interior. A large fraction of these movements depend on microtubules, tiny hollow tubes that are found throughout the cytoplasm. Microtubules serve either as tracks to transport cargo such as organelles, vesicles, proteins and RNA, or as ropes on which motor proteins pull in order to move structures such as the mitotic spindle, centrosome and the nucleus (Pavin and Tolić-Nørrelykke 2013; Franker and Hoogenraad 2013; Tolić-Nørrelykke 2008). In order to perform these functions, microtubules must first target their cargo, or regions in the cell where they can load the cargo, or cortical anchor sites for the motors to pull on them. A key question is how microtubules find their targets.

The most prominent example of microtubule-driven movement is the segregation of chromosomes into the two future daughter cells during cell division. At the onset of division, the cell forms a spindle, an accurate self-constructed micro-machine, in which vital parts are microtubules that connect the spindle poles with the chromosomes, and pull on chromosomes to divide the genetic material between the two nascent daughter cells (Cheeseman and Desai 2008). The attachment of microtubules to chromosomes is mediated by kinetochores, protein complexes on the chromosome.

To understand the mechanisms of how microtubules find kinetochores, one needs to know their properties. Microtubules are dynamic polymers that grow or shrink, where a switch between these states occurs in a stochastic manner. In mitotic animal cells, microtubules nucleate mainly from centrosomes, whereas in plants and meiotic cells that lack centrosomes, microtubules nucleate around chromosomes. A variety of microtubule-associated proteins regulate the dynamics and nucleation of microtubules (Akhmanova and Steinmetz 2010; Teixido-Travesa et al. 2012). The kinetochore is a protein complex which is very sticky: when it encounters a microtubule, a stable connection is formed between them (Mitchison and Kirschner 1985). The kinetochore size and composition varies between different cell types, as well as the microtubule properties. Thus, different cell types rely on different mechanisms of kinetochore capture. Several mechanisms have been proposed based on experiments, and some of them have been tested theoretically. Here we review the concepts behind these mechanisms with emphasis on the recently discovered microtubule property to explore the space by pivoting around the spindle pole (Kalinina et al. 2013). Finally, we discuss how pivoting helps microtubules to find various targets in different cellular contexts.

## Mechanisms of kinetochore capture

### Search-and-capture

**Fig. 1:**
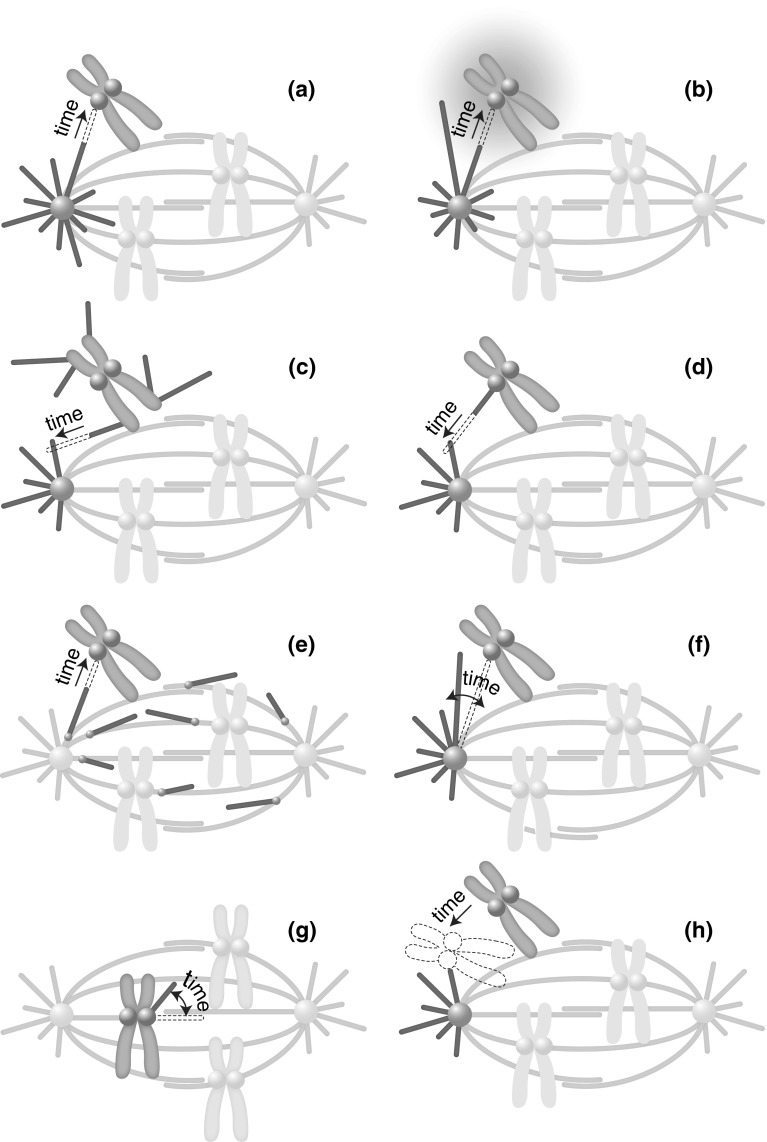
Models of kinetochore capture. **a** Search-and-capture; **b** bias in microtubule dynamics towards the chromosomes; **c** nucleation of microtubules at the chromosomes; **d** nucleation of microtubules at the kinetochores; **e** nucleation of microtubules at spindle microtubules; **f** pivoting of microtubules around the spindle pole; **g** pivoting of kinetochore-bound microtubules; **h** kinetochore movements. Microtubules are depicted as *green lines*, kinetochores as pink spheres on the chromosomes (*gray*), and centrosomes as *gray spheres*. Ran-GTP is represented by a *pink* *gradient* in (**b**), and augmin complexes by small blue spheres in (**e**). *Dashed lines* mark microtubule growth (**a**–**e**), microtubule pivoting (**f**, **g**) and the movement of the chromosome (**h**). In *each panel*, structures of interest are intensely colored, whereas the remaining parts of the spindle are pale. (Color figure online)

The pioneering idea termed “search-and-capture” relies on the dynamics of microtubules and their nucleation at centrosomes (Kirschner and Mitchison 1986). As a microtubule grows from the centrosome in an arbitrary direction, it probes the space as it searches for kinetochores. Even though a single microtubule probes only one direction, numerous directions will be explored eventually because numerous microtubules grow from the centrosome (Fig. 1a).

In the first theoretical study that investigated the search-and-capture model, the average capture time was calculated for numerous microtubules searching for a single target at a certain distance (Hill 1985). A similar study has shown that the capture time depends strongly on the distance between the kinetochore and the centrosome, and for a typical distance of 10 μm it takes ten(s) of minutes to capture a single kinetochore (Holy and Leibler 1994). In case of a system with multiple kinetochores, the time needed to capture all kinetochores grows logarithmically with the number of kinetochores (Wollman et al. 2005; Gopalakrishnan and Govindan 2011). Thus, the search-and-capture model predicts that in a human cell, which has 46 chromosomes, it would take roughly 100 min to capture all kinetochores. However, human cells capture their kinetochores within 20 min (Wollman et al. 2005), which is five times faster than the theoretical prediction.

### Bias in microtubule dynamics towards the chromosomes

As the search-and-capture model predicts a slower kinetochore capture kinetics than that measured in various cell types, several mechanisms have been proposed to accelerate the search for kinetochores (Duncan and Wakefield 2011; Mogilner and Craig 2010; O’Connell and Khodjakov 2007). One of the reasons for the slow kinetics of capture in the search-and-capture model is that the majority of the microtubules grow in futile directions, towards empty regions of the cell devoid of chromosomes. If the microtubules would “know” where the chromosomes are, this would speed up their search.

To signal their location in the cell, chromosomes create a spatial gradient based on the GTPase Ran (Clarke and Zhang 2008). When bound to GTP, Ran stabilizes microtubules and promotes spindle assembly in *Xenopus laevis* egg extracts (Zhang et al. 1999; Ohba et al. 1999; Wilde and Zheng 1999; Carazo-Salas et al. 1999; Kalab et al. 1999). The exchange of GDP for GTP on Ran is promoted by its guanine-nucleotide-exchange factor RCC1 (Bischoff and Ponstingl 1991). As RCC1 is bound to the chromosomes, a high local concentration of Ran-GTP is generated at the chromosomes (Carazo-Salas et al. 1999; Kalab et al. 2002). Ran-GTP can diffuse away from the chromosome before the GTP becomes hydrolyzed into GDP, thereby forming a gradient of Ran-GTP that decreases with distance from the chromosomes.

What aspects of microtubule dynamics does the Ran-GTP gradient affect, leading to a more efficient kinetochore capture? Ran-GTP provides a spatial signal that influences microtubule dynamics at a distance from the chromosome, by increasing microtubule rescue frequency and decreasing catastrophe frequency (Carazo-Salas et al. 2001; Wilde et al. 2001). Thus, microtubules that extend into a region close to a chromosome, where the Ran-GTP concentration is high, continue growing, leading to an asymmetric distribution of microtubules with a bias towards the chromosomes (Fig. 1b) (Carazo-Salas and Karsenti 2003).

A theoretical study has explored a biased search-and-capture in the presence of a spatial gradient around the kinetochore of a stabilizing factor that inhibits microtubule catastrophe, thus microtubules that extend towards the kinetochore grow long enough to reach the kinetochore. This bias in microtubule dynamics speeds up the capture of kinetochores (Wollman et al. 2005). Such a bias might be more important for kinetochore capture and spindle assembly in early embryonic systems than in somatic cells, because the cells in early developmental stages are large and may rely on positional cues for microtubule growth more than the smaller somatic cells.

### Nucleation of microtubules at the chromosomes and kinetochores

The search-and-capture model relies on the microtubules nucleated at the centrosome, thus this model is also called the centrosomal pathway. Yet, microtubules may be nucleated at additional locations. Indeed, it has been shown that chromosomes influence the local assembly of microtubules during metaphase in animal cells, which is known as the chromosomal pathway (Karsenti et al. 1984). Similarly to the stabilization of microtubules in the vicinity of chromosomes, nucleation of microtubules at the chromosomes is also promoted by Ran-GTP (Carazo-Salas et al. 1999). Interactions between chromosomal and centrosomal microtubules may help spindle assembly (Fig. 1c).

Kinetochores are also able to nucleate microtubules, both on isolated mitotic human chromosomes (Telzer et al. 1975) and in mammalian cells in vivo (Witt et al. 1980; De Brabander et al. 1981). Analogously to the microtubules nucleated around the chromosomes, kinetochore-nucleated microtubules may speed up kinetochore capture and spindle assembly (Fig. 1d) (Tulu et al. 2006; Maiato et al. 2004).

On a fully-formed metaphase spindle, kinetochores are attached to the plus ends of microtubules (Euteneuer and McIntosh 1981). However, microtubules are generally nucleated from the minus end. What is then the polarity of microtubules that are nucleated at kinetochores? Mammalian and yeast kinetochores have been shown to nucleate microtubules with distal plus ends, just like the centrosomes do (Bergen et al. 1980; Kitamura et al. 2010). Thus, the kinetochores must switch from having proximal minus ends to the proximal plus ends. Upon interaction of kinetochore-nucleated microtubules with the centrosomal ones, the kinetochore microtubules disassemble and the kinetochore remains attached to the centrosomal microtubule. This microtubule has the “correct” polarity with the plus end towards the spindle midzone, allowing the kinetochore to eventually reach the plus end (Kitamura et al. 2010).

The role of microtubules nucleated at kinetochores has been explored in a theoretical study. Computer simulations have shown that the presence of microtubules of a fixed length nucleated at the kinetochores accelerates kinetochore capture, because these microtubules extend the target area that the dynamically unstable centrosomal microtubules must hit (Paul et al. 2009).

### Nucleation of microtubules at spindle microtubules

In addition to microtubule nucleation at the centrosome and the chromosomes, microtubules are nucleated along the pre-existing ones in the spindle (Fig. 1e). These microtubules, which grow parallel or almost parallel to the template microtubule, may participate in the process of kinetochore capture by interacting with a kinetochore, thereby connecting it to the template microtubule. The process of microtubule nucleation throughout the spindle has been observed in cells without and with centrosomes, and it has been shown that it depends on the augmin complex (Burbank et al. 2006; Mahoney et al. 2006; Goshima et al. 2008).

### Microtubule pivoting around the spindle pole

A common feature of the mechanisms described above is that microtubules, as they grow, probe the space by their tips. On the contrary, if a microtubule moves laterally it can probe the space by its entire length (Fig. 1f). In this case, the efficiency of the search does not depend on the microtubule growth velocity, but instead on the microtubule length and on how fast the microtubule moves laterally.

In the spindle, where microtubules must link two binding partners, a centrosome and a kinetochore, it is puzzling how a microtubule that is with one end bound to one binding partner can explore the space laterally, as it searches for the other partner. In principle, lateral movement is possible if the bound end of the microtubule is freely jointed to a fixed point, thus allowing the microtubule to pivot around this point. In fission yeast, a microtubule that is bound to the spindle pole body changes its orientation with respect to the cell (Kalinina et al. 2013). In other words, the microtubule pivots around the spindle pole body, swiping through the cell and exploring the space laterally in search for kinetochores, until it approaches and captures a kinetochore. This pivoting movement is random and most likely driven by thermal forces because it does not require ATP. The pivoting can be characterized by an angular diffusion coefficient, which for a 1.5 μm-long microtubule has a value of *D* = 0.001 rad^2^/s. This angular diffusion can explain the measured typical time of kinetochore capture, demonstrating that the search for kinetochores is driven by microtubule pivoting (Kalinina et al. 2013).

Pivoting is not restricted to the microtubules growing from the spindle pole. In *Drosophila* S2 cells, microtubules extending from the kinetochore have been observed to pivot (Fig. 1g) (Maiato et al. 2004). These kinetochore-bound microtubules are initially not oriented towards a spindle pole, but they change their orientation while growing and eventually become captured by the microtubules growing from the centrosome. Similarly, after laser cutting of a microtubule that connects a kinetochore with the centrosome, the microtubule stub that remains associated with the kinetochore pivots as it grows, ultimately becoming integrated into the spindle (Maiato et al. 2004).

### Kinetochore movements

In all the mechanisms described above microtubules probe the space as they search for kinetochores or other microtubules. However, it could be the other way around: by moving around, the kinetochore may probe the space in search for microtubules (Fig. 1h).

The role of kinetochore movements, which include diffusive motion and rotations, in the process of kinetochore capture was studied in human cancer cells (Paul et al. 2009). Experiments have shown that kinetochores, while being associated with the chromosome, perform diffusive movement through the cytoplasm with a diffusion coefficient of *D* = 0.01 μm^2^/s. The theory, which is based on the measured diffusion of kinetochores and the known properties of microtubules, shows that kinetochore diffusion and rotations accelerate spindle assembly including capture of kinetochores (Paul et al. 2009). Diffusion of kinetochores was measured also in fission yeast, where its value is lower, *D* = 0.0006 μm^2^/s, and its contribution to the capture process is minor (Kalinina et al. 2013).

As described in this section, numerous mechanisms of kinetochore capture have been identified, but one mechanism does not exclude contributions of others. Thus, a systematic theoretical approach that takes into account all mechanisms is needed to understand the process of kinetochore capture. The results depend on the microtubule and kinetochore characteristics included in the model, and thus can differ between various cell types. For example, a single mechanism may be sufficient to explain the kinetics of kinetochore capture in yeast (Kalinina et al. 2013). Alternatively, theory may reveal that the typical capture time can be reproduced only by including several mechanisms, which was shown in a study on human cells (Paul et al. 2009).

## Functions of microtubule pivoting in various cellular situations

### Spindle translocation: Pivoting promotes cortical capture of microtubules

During mitosis in budding yeast, the spindle is formed inside the mother cell and has to move through a narrow neck into the daughter cell or the bud, in order to segregate the chromosomes equally between the two cells. Astral microtubules, which grow from the spindle poles, are the main players that move the spindle via their interactions with the cell cortex. Two types of motor proteins are involved in the generation of force on the microtubules and thus on the spindle: myosin V and cytoplasmic dynein (Yin et al. 2000; Eshel et al. 1993; Li et al. 1993). Myosin V, which is bound to the plus end of the microtubule via the adaptor protein Kar9 (Miller and Rose 1998), walks along the actin cables at the cell cortex, thereby moving the plus end along the cortex. As the actin cables in the mother cell are polarized towards the neck, the plus end eventually reaches the neck. As the pus end is moving, the whole microtubule performs angular movement (Liakopoulos et al. 2003). This movement of the microtubule results in the angular movement of the spindle, which follows the microtubule, becoming oriented towards the neck. Thus, the angular movement of microtubules is important for the orientation of the spindle towards the neck.

**Fig. 2:**
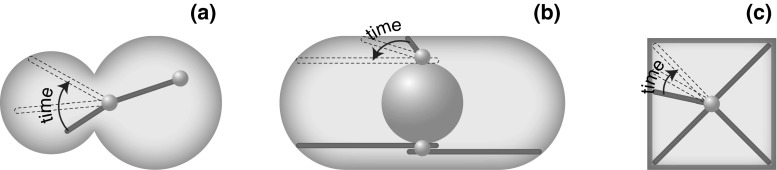
Microtubule pivoting in various cellular contexts and in vitro. **a** Pivoting promotes cortical capture of astral microtubules during spindle translocation from the mother to the daughter cell in budding yeast. **b** Pivoting helps the formation of a parallel array of microtubule bundles in interphase fission yeast cells. **c** Pivoting helps microtubules to center the microtubule aster in a microfabricated chamber. *Green lines* represent microtubules; *small gray* *spheres* depict the spindle pole bodies in (**a**), microtubule-organizing centers in (**b**) and the centrosome in (**c**); the *large gray sphere* in (**b**) is the nucleus. *Dashed lines* mark different positions of the microtubules as they pivot and explore the space laterally, searching for their targets. (Color figure online)

Once the spindle is near the neck, astral microtubules enter the daughter cell. To pull the spindle through the neck, dynein that is on the microtubules needs to be off-loaded to the cortical anchor protein Num1 (Lee et al. 2005). Angular movement of microtubules, driven by myosin V, most likely helps the microtubules to reach a cortical anchor for dynein (Baumgartner 2011). Thus, the angular movement of microtubules promotes not only the orientation of the spindle towards the neck inside the mother cell, but also the cortical capture of microtubules inside the daughter cell (Fig. 2a), to allow for the interaction with cortical anchors, which is needed for dynein to exert force on the microtubule.

### Interphase microtubules: Pivoting helps the formation of a parallel array of microtubule bundles

Microtubule pivoting is important for the generation of self-organized microtubule structures. For example, the rod-shaped fission yeast cells display several microtubule bundles that lie parallel to the long axis of the cell during interphase. Each bundle consists of typically two antiparallel microtubules with their minus-end regions bundled together near the nucleus and the plus ends pointing towards the cell tips (Drummond and Cross 2000). The alignment of the microtubules is crucial for correct positioning of growth sites because the microtubule plus ends deposit polarity factors to the cell tips, thereby restricting the growth pattern of these cells to extension in length at the cell ends (Mata and Nurse 1997; Martin et al. 2005; Tatebe et al. 2005; Baumgartner and Tolić-Nørrelykke 2009). Moreover, the alignment of microtubules during interphase determines the initial alignment of the mitotic spindle (Vogel et al. 2007) and their position determines the position of the nucleus and of the cell division plane (Tolić-Nørrelykke et al. 2005; Daga and Chang 2005; Tolić-Nørrelykke 2010).

How do the microtubule bundles become aligned with the cell axis? Live-cell imaging has shown that microtubules grow from the central region of the cell. Those that contact the cell cortex at an oblique angle, change their angle as they continue growing towards the cell tip and eventually become parallel to the longitudinal axis of the cell (Brunner and Nurse 2000). Thus, through their angular movement, microtubules target specific location in the cell, in this case the cell tips (Fig. 2b). This mechanism of targeting requires regulation of microtubule dynamics, such that the microtubules do not undergo catastrophe too soon after contacting the cell cortex on the lateral sides of the cell, and also not too late after contacting the cortex at the cell tips, which would result in their curling around the cell tips. The regulation of microtubule dynamics is achieved by the proteins that suppress catastrophe, such as the CLIP-170 homolog Tip1 (Brunner and Nurse 2000) and the EB-1 homolog Mal3 (Beinhauer et al. 1997).

### Microtubule asters in microfabricated chambers: Pivoting helps microtubules to center the aster

In animal cells, the cleavage plane is determined by the position of the spindle, which in turn is governed by microtubules emanating from the two centrosomes, forming two microtubule asters. Microtubules interact with their plus ends with the cell cortex, where cortical dynein pulls on the microtubule (Gonczy et al. 1999; Grill et al. 2003). In fission yeast, dynein reaches cortical anchor sites by diffusing along the microtubule (Ananthanarayanan et al. 2013), and detaches from the cortex in response to load forces (Vogel et al. 2009). An important question is how dyneins, by pulling from the cortex, position the microtubule aster within a confined geometry of the cell.

The process of positioning of microtubule asters can be studied in vitro in a microfabricated chamber. Such approach is ideally suited for investigation of the positioning mechanisms, because one can identify a minimal number of components that are necessary to mimic positioning within cells. A study in which microtubule asters were grown within the chambers with walls coated by dynein motors showed that microtubules are distributed in an anisotropic manner, being preferentially oriented towards the boundaries distal from the centrosome (Laan et al. 2012). Such anisotropy occurs when the tip of the growing microtubule slips along the boundary. As the tip slips, it travels towards the distal boundaries and drives microtubule pivoting that reorients the microtubule towards the distal boundaries (Fig. 2c). Finally, if there is pulling from the cortex, the net force on the anisotropic distribution of microtubules is oriented towards the center of the chamber. Thus, pivoting of the microtubules around the centrosome, as they are becoming reoriented towards the distal boundaries, is crucial for the centering of the centrosome.

## Conclusion and outlook

An important challenge for future work will be to unravel the molecular basis of the free joint that allows for pivoting of the microtubules. Pivoting has been observed in yeast, where the joint is at the spindle pole body, and in higher eukaryotic cells, where microtubules pivot around the centrosome. Although the molecular architecture of the spindle pole body differs from that of the centrosome, the angular movement of the microtubules growing from these two structures seems to be similar. Moreover, microtubules exhibit angular movement also when growing from kinetochores. It will be important to identify the molecules building up the free joint in cases where the microtubule is attached to different cellular structures.

Microtubule pivoting is a major mechanism underlying the search for kinetochores in fission yeast. A combination of experiments and theory has shown that microtubule pivoting speeds up kinetochore capture roughly 30 times compared to a situation in which the microtubules only grow and shrink but do not pivot (Kalinina et al. 2013). It will be interesting to explore to which extent microtubule pivoting contributes to the search for different targets in different cellular settings.
